# Supplementary material for: Identification of photoperiod-induced specific miRNAs in the adrenal glands of Sunite sheep (Ovis aries)
Source: Front Vet Sci. 2022 Jul 22;9:888207. doi: 10.3389/fvets.2022.888207 (PMC9354845; doi:10.3389/fvets.2022.888207)
Supplement: Supplementary file 1 [file Data_Sheet_1.ZIP › Supplementary file/miRDeep2 file.zip!/miRDeep2 file/Novel_Structure/LP42A3/Novel_20.pdf]

Provisional ID	:	NC_019468.2_13163
Score total	:	2
Score for star read(s)	:	-1.3
Score for read counts	:	0
Score for mfe	:	1.7
Score for randfold	:	1.6
Score for cons. seed	:	
Total read count	:	18
Mature read count	:	18
Loop read count	:	0
Star read count	:	0

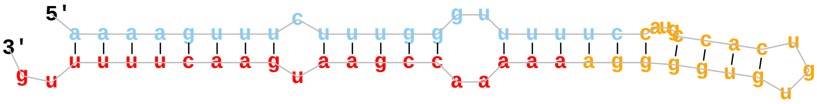

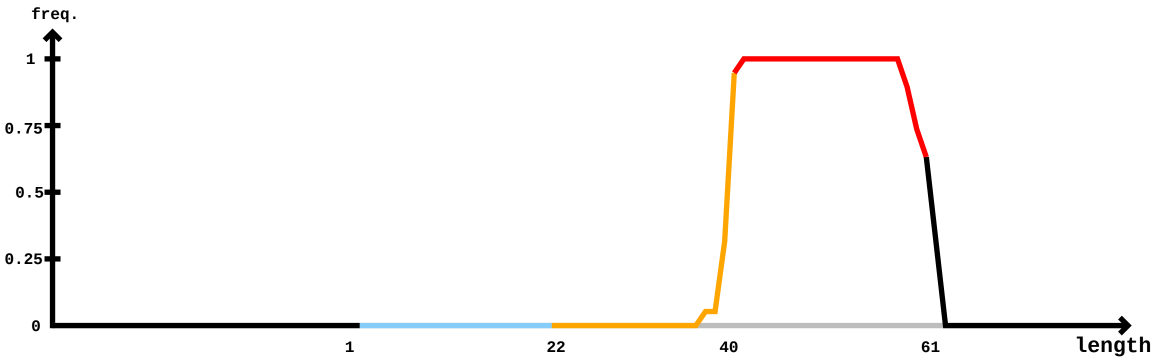

Star Mature

```
5'- caguggaaguaauuuguauuggguugaccaaaaaguuucuuuggguuuuuccaugccacugugugggaaaaaaccgaaugaacuuuuuggccaacacaauauuuucuuu   -3'    exp
    ....(((((......((((((.((((.(((((((((((.(((((..((((((...((((...))))))))))..))))).))))))))))).)))).)))))))))))..   reads      mm         sample
    ..................................................................ggaaaaaCccgaaugaacuuu.......................   1          1          seq
    ....................................................................aaaaaaccAaaugaacuuu.......................   1          1          seq
    ....................................................................aaaaaCccgaaugaacuuuu......................   2          1          seq
    ....................................................................aaaaaUccgaaugaacuuuuu.....................   1          1          seq
    ....................................................................aaaaaCccgaaugaacuuuuu.....................   1          1          seq
    .....................................................................aaaaUccgaaugaacuuuu......................   1          1          seq
    .....................................................................aaaaUccgaaugaacuuuuug....................   1          1          seq
    .....................................................................aaaaCccgaaugaacuuuuug....................   6          1          seq
    .....................................................................aaaaUccgaaugaacuuuuugg...................   4          1          seq
    ......................................................................aaaaccgaaugaacuuuuug....................   1          0          seq
```
